# Functional Genomics in Psoriasis

**DOI:** 10.3390/ijms25137349

**Published:** 2024-07-04

**Authors:** Stefano Rossi, Ellie Louise Richards, Gisela Orozco, Stephen Eyre

**Affiliations:** Centre for Genetics and Genomics versus Arthritis, Division of Musculoskeletal and Dermatological Sciences, Faculty of Biology, Medicine and Health, University of Manchester, Manchester M13 9PL, UK; stefano.rossi@manchester.ac.uk (S.R.); ellie.richards@manchester.ac.uk (E.L.R.); gisela.orozco@manchester.ac.uk (G.O.)

**Keywords:** psoriasis, functional genomics, GWAS, inflammation, precision medicine

## Abstract

Psoriasis is an autoimmune cutaneous condition that significantly impacts quality of life and represents a burden on society due to its prevalence. Genome-wide association studies (GWASs) have pinpointed several psoriasis-related risk loci, underlining the disease’s complexity. Functional genomics is paramount to unveiling the role of such loci in psoriasis and disentangling its complex nature. In this review, we aim to elucidate the main findings in this field and integrate our discussion with gold-standard techniques in molecular biology—i.e., Clustered Regularly Interspaced Short Palindromic Repeats (CRISPR)—and high-throughput technologies. These tools are vital to understanding how disease risk loci affect gene expression in psoriasis, which is crucial in identifying new targets for personalized treatments in advanced precision medicine.

## 1. Definition and General Understanding of Psoriasis

Psoriasis is a noncommunicable, chronic, inflammatory, immune-mediated disease affecting 0.5–4.6% of the global population [1]. The disease is characterised by keratinocyte overgrowth and immune infiltration and presents in different forms including guttate, erythrodermic, inverse, pustular, and palmoplantar psoriasis [2]. The most common form is plaque psoriasis or Psoriasis Vulgaris (PV), accounting for nearly 90% of cases [3], and presenting with scaly, thick skin lesions typically on the nails, scalp, genitals, joints, lumbosacral area, and buttocks [4]. The disease has equal prevalence among genders and a bimodal onset distribution, peaking around the ages of 18–39 and 50–69 [5]. While psoriasis is rare in children—ranging from 0% in Taiwan to more than 2.1% in Italy [5]—the incidence rates are more variable in adults and have increased over time [6], with a higher prevalence in white people (3.6%) than in African Americans (1.5%) and Hispanics (1.9%) [7]. The clinical severity of such a skin ailment varies significantly over time, with cyclical periods of flares that are followed by subsidence or remission [2]. Importantly, psoriasis involves both the innate and adaptive branches of the immune system and involves the aberrant production of proinflammatory molecules. This leads to prolonged harm to several tissues and systemic inflammation [2]. Co-morbidities, including psoriatic arthritis (PsA), metabolic problems, anxiety, psychosocial distress, depression, and inflammatory bowel disease, are common among individuals with psoriasis, significantly impacting their quality of life [4].

Despite the array of treatments available in psoriasis—e.g., biologics [8]—these interventions do not cure the disease, and not all patients respond appropriately. Thus, there remains an unmet need for therapies that could permanently halt rather than manage clinical symptoms. As described in Table 1, we aim to highlight different aspects of psoriasis while providing a comprehensive outline of the complexity of this disease.

**Table 1:** Summative table of sections outlined in this review.

Section	Key Message	References
Definition and genetics of psoriasis	Psoriasis is a multifactorial, autoinflammatory, dermatological condition characterised by raised scaley lesions across the body. Whilst influenced by environmental and lifestyle factors, it has a large genetic component. Variants in PSORS1, located in the HLA-C gene, have been identified as the main genetic risk factor for psoriasis, accounting for 30–50% of disease heritability.	[2,3,4,5,9]
GWAS in psoriasis: benefits and limitations	The first large-scale GWAS in psoriasis by Cargill et al. confirmed the importance of IL12B and IL23R. Both are now used successfully as targets for treating psoriasis. More recent GWASs, such as the meta-analysis by Dand et al., have identified even more unique signatures. However, GWASs cannot assign a causal variant, gene, or cell type or elucidate the biological mechanism driving the SNP–phenotype relationship.	[10,11]
Post-GWAS analysis of psoriasis-associated SNPs using functional genomics	Techniques such as fine mapping, epigenetic analysis, chromatin conformation capture and eQTL analysis can reveal the structural context of genetic changes and identify physical interactions between genetic landmarks. Additionally, by identifying SNPs in cell type-specific enhancers, the causal cell types driving psoriasis phenotypes can be identified.	[12,13,14,15]
Examining phenotypic differences using advanced functional techniques	The revolutionary gene-editing technique CRISPR can be utilised to activate, repress, delete or alter a region of interest to assess the genetic and phenotypic consequences of suspected lead variants in psoriasis. This technique can be applied on the cellular level—both in cell lines and primary cells—as well as in organoid systems and whole organisms such as mouse models.	[16,17,18,19,20,21]
Towards novel psoriasis therapeutics	Drug repurposing utilises treatments that have already been used to treat other diseases. This method dramatically speeds up development time and reduces costs, as safety and pharmacodynamic profiles are already known for these drugs. Examples include the holistic treatment Esculetin and cancer drugs targeting POLI and IL-13. AI can also be used to speed up this process. While CRISPR-Cas9 has been used to treat sickle-cell disease and transfusion-dependent β-thalassemia, it has not yet been applied to psoriasis. Wan and colleagues suggest that the first RNP treatment for psoriasis could be on the horizon.	[22,23,24,25,26,27]

## 2. The Significant Genetic Component in Psoriasis

The aetiology of psoriasis is multifactorial. This complex disease is influenced by environmental factors such as skin injury, UV exposure, immune-modulating drugs, smoking, dietary habits, alcohol, microbiota, obesity, and stress [28]. However, these factors alone do not fully account for the risk of developing psoriasis. Genetic epidemiology studies have highlighted the highly heritable pattern of psoriasis, pinpointing its strong genetic component [29,30]. Linkage studies in families, measuring vertical allele transmissions through generations, and genetic association studies, assessing different allele frequencies between cases and controls, have unequivocally confirmed the genetic roots of psoriasis [31,32]. Specifically, linkage studies demonstrated 15 psoriasis susceptibility (PSORS) loci [33,34]. Among these, PSORS1 has been identified as the main genetic factor influencing the risk of developing the disease and is located within the major histocompatibility complex region. Notably, PSORS1 accounts for circa 35–50% of psoriasis heritability [9]. The causal variant for psoriasis was identified through deep sequencing as HLA-C*06:02 [35], which is found in 20–50% of psoriasis patients and is linked with various disease characteristics [36,37]. Although traditional linkage analysis has contributed to uncovering the genetic basis of psoriasis, recent advances have harnessed the power of large case and control cohorts, genotyped with single nucleotide polymorphism (SNP) microarrays.

GWASs identify connections between genotypes and phenotypes, enabling testing for SNP allele frequency variations among groups of individuals with a trait compared to controls (Table 2). GWAS screens identify clusters of correlated SNPs, each associated with a particular trait and described as genomic risk loci [38].

The first GWAS was published in 2005 on macular degeneration [39]. Since then, over 5700 GWASs have explored more than 3300 traits [40], with sample sizes exceeding a million participants. Complex traits are affected by a substantial number of causal variants—each contributing modestly to the overall risk [38,41]. However, these variants are highly correlated with many other SNP variants in high linkage disequilibrium (LD) and often map to non-coding parts of the genome [42]. As a result, a disease-associated variant does not necessarily provide insights into the involved gene/s, causal variant/s, or the exact mechanism by which such variation influences phenotypic modifications [43]. 

GWASs have revolutionised the study of complex, autoimmune diseases and have been further complemented with targeted studies—e.g., Immunochip by Illumina [44]. These studies have made it possible to discover genetic connections across different autoimmune conditions [43]. Since 2007 [10], several large scale GWASs across multiple populations, including in East Asia and Europe, have identified more than 80 psoriasis-associated loci [45].

**Table 2:** Brief description of techniques outlined in this review.

GWASs	GWASs determine associations between different genotypes and phenotypes to identify clusters of correlated SNPs associated with a particular trait [38]. Such screens can identify new genetic markers that increase susceptibility to psoriasis.
ATAC-seq	ATAC-seq determines transcriptionally active, open chromatin regions [46]. Changes in chromatin accessibility in psoriasis offer clues into potentially critical genome spots in disease susceptibility.
ChIP-seq	ChIP-seq identifies histone modifications in targeted genomic regions, pinpointing the biological roles of epigenetic markers in different conditions or diseases [47]. In psoriasis, the ChIP-seq method can identify unknown gene regulatory mechanisms to identify novel therapeutic approaches.
Capture Hi-C (CHi-C)	CHi-C identifies specific regions—e.g., promoters or enhancers—through a hybridisation step, enriching and increasing the resolution of these areas of interest [48] compared to classical chromosome conformation capture techniques. Psoriasis-related long-range interactions can identify DNA rearrangements that could increase the risk of developing the disease.
CRISPR	The CRISPR system is a powerful tool that classically cuts targeted DNA sequences with breaks prone to alteration, deletion, or addition of genetic sequences [16]. This technique can help study genes or non-coding regions involved in psoriasis.

## 3. GWASs in Psoriasis

Cargill and colleagues performed the first large-scale GWAS in psoriasis, which confirmed the importance of the *IL12B* and *IL23R* genes in disease risk [10]. It included 1432 controls and 1446 cases of European descent, and more than 25,000 gene-centric SNPs [10]. Confirming this connection between the IL-12 pathway—specifically, the *IL12B* variants—and psoriasis was a study performed in a Japanese cohort [49]. Several psoriasis GWASs were then pooled in a meta-analysis [50] and a drug-repositioning analysis was performed, leading to the identification of seven drug target genes from six different newly identified loci for 18 potential drugs that are currently employed in clinics to treat the disease [12]. A study on a Japanese psoriasis cohort was carried out in 2016 by Nishikawa and colleagues. Their study focused on how patients responded to anti-TNF-α therapy, correlating SNP variants with positive treatment outcome and implicating *JAG2* and *ADRA2A* in the response to treatment [51]. Two years later, the largest GWAS on PV within a Japanese cohort [52] underlined the complex architecture of psoriasis while corroborating findings from the previously published European study describing the pivotal role of *TNIP1*—an inflammatory marker dysregulated in several autoimmune conditions—in disease susceptibility [52]. The most recent psoriasis meta-analysis combined data from 18 GWASs and included over 35,000 cases and 450,000 controls. The study added 45 novel psoriasis risk loci, taking the total to 109 loci. This study confirmed the importance of the IL-23 pathway in the disease, with *IL23R*, *IL23A*, *IL12B*, and *STAT3* all strongly implicated in disease risk, which correlates with the success of the biologic pathways targeted in therapies [11].

## 4. Limitations and Potential Benefits of GWASs in Understanding Psoriasis

There have been tremendous advances in the understanding of complex diseases through GWASs [53,54]. In psoriasis, these include the 109 genetic loci robustly associated with increasing the risk of disease [11], the implication of the IL-23/Th17 pathways in the disease [55], the genetic overlap between psoriasis and Crohn’s disease [56], the association with the class 1 *HLA* locus and *ERAP1* [57], implicating the trimming and presenting of peptides as a key factor in the disease, the overlap in genetic susceptibility between ethnic groups and the confirmation that the disease is driven by a genetically altered immune and skin system. There are, however, limitations to GWAS findings [58,59,60,61]. Due to the nature of the associated risk alleles—which are highly correlated with a number of other alleles and mainly found outside protein coding regions—it is not trivial to assign a causal variant, causal gene, causal cell type or mechanism to the GWAS signals. If these are better defined, there is the possibility to improve the translation of genetic findings into the clinic. To date, translation involves highlighting potential novel drug targets, e.g., TYK2 in psoriasis [62]; re-positioning currently available therapeutics, e.g., targeting the IL-23 pathway used in Crohn’s and psoriasis [63]; generating genetic risk scores for patients dependent on which biological pathways are most genetically perturbed and relating this to treatment-response and other clinical outcomes; and the pinpointing of novel genetic therapeutics, such as the CAR-T cell therapy including cancer, autoimmune disorders, and infections [64,65]. 

## 5. Post-GWAS Analysis of Psoriasis-Associated SNPs

Fine mapping is a technique commonly used to refine the signals from GWASs, determining which SNPs are more likely to be causal for a given trait or disease. A variety of techniques for fine mapping have been developed, ranging from single to multiple causal variant mapping. Single variant mapping is typically accomplished using a Bayesian method, testing multiple hypotheses and generating a credible set of potential variants [13]. These credible sets can then be narrowed down to an adjusted credible set by using the conditional coverage estimate, claimed coverage, and adjusted coverage estimate [14]. 

Although single causal variant fine mapping does not accurately represent biological mechanisms, it does provide a framework for more complex mapping [13], such as that used by Dand and colleagues in the latest psoriasis-specific GWAS meta-analysis [11]. This study used a stepwise model and modified Bayesian statistical fine mapping to analyse eighteen GWASs of European ancestry in a meta-analysis. The strongest association (rs12189871) was found in the HLA-Cw6 region, previously labelled as the PSORS1 loci [66], accounting for 35–50% of disease heritability within psoriasis [67]. 

Fine mapping can provide a statistically prioritised list of the most likely causal variants, providing more information than be gained from the ‘lead GWAS’ SNP, where it has been estimated that as little as 5% of lead SNPs identified in GWASs are causal and can actually be some distance from the true causal SNP [68]. Therefore, GWASs and statistical fine mapping are not sufficient to identify the causal SNPs, genes and mechanism by which SNPs cause disease and must be integrated with functional genomics to maximise the genetic discoveries.

## 6. The Use of Functional Genomics in Psoriasis Research

Functional genomics comprises numerous high-throughput experimental techniques that offer valuable insights into the functional components of the genome, including but not limited to gene expression, chromatin accessibility, DNA methylation, and protein–DNA interactions. They reveal the dynamic transcriptional changes caused by SNPs in disease-specific contexts [15].

Coding SNPs within the exons of genes are generally easier to interpret as they can alter protein sequences, splicing and function. Therefore, they directly influence disease phenotypes [22]. For example, the SNP rs148755083 located within the *IL36RN* gene generates a homozygous missense mutation of the IL-36 receptor agonist, increasing the likelihood of developing psoriasis via abnormal interleukin signalling [69,70]. However, approximately 90% of disease-associated SNPs are in non-coding regions, such as introns, enhancers and promoters, presenting a challenge in determining their functional significance [68,71]. 

Of these non-coding disease risk SNPs, approximately 60% lie within enhancers and specialized regions with a strong propensity for binding transcription factors [68]. Each cell type has its own specific gene expression patterns, which are directly controlled by cell- and tissue-specific enhancers and differential master transcription factors [72]. Therefore, it is crucial to understand the impact of these SNPs in the relevant cell types and in the context of the disease. 

**Figure 1:**
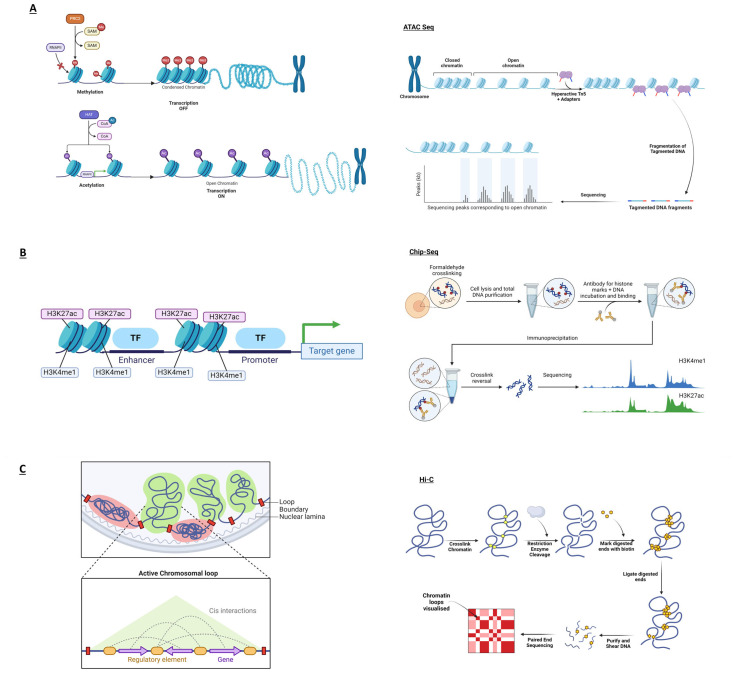
Overview of techniques in functional genomics. (**A**) Left panel: Tightly packed chromatin, marked by histone methylation and mediated by methyl group transfers and a group of proteins which modify chromatin, prevents the transcriptional machinery from accessing the DNA molecule, making the latter transcriptionally inactive. On the other hand, upon an enzyme-mediated histone acetylation, chromatin loosens and facilitates RNA polymerase II (RNAPII) to start transcription. Loosened chromatin regions can be studied using the ATAC-seq technique. Right panel: The Tn5 transposase catalyses the integration of adapters into accessible DNA segments—fragmenting DNA. This product can then be sequenced to pinpoint peaks of open, active, chromatin regions. (**B**) Left panel: The study of chromatin state is crucial in regulating gene expression. Right panel: The ChIP-seq technique determines epigenome marks (such as enhancers) and several epigenetic signatures. DNA and proteins form cross-links in formaldehyde-fixed cells. Upon cell disruption, extracted DNA is then tagged with histone marker-specific antibodies to determine cross-linked DNA–protein interactions. Cross-linked DNA is then released and then sequenced. (**C**) Left panel: The nature of chromatin packing influences gene regulation, with active loops (in green) being transcriptionally active and inactive loops (in red) suppressing it. Right panel: The preserved chromatin structure is processed with restriction enzymes with biotin-tagged ends. Subsequently, ligated ends are then purified and sequenced to identify interactions across distant genomic regions. Images generated with BioRender.com.

For a region of DNA to become an active gene regulatory element, it must unwind from the proteins (histones) that keep it compacted and become open and active (Figure 1). These histones are then modified, for example, through methylation or acetylation, to maintain the DNA in an open and active conformation. Open chromatin can be measured in cells through directed sequence reactions—that is, the ATAC-seq technique [46], and modified histones can be measured through antibody enrichment of the DNA by performing the ChIP-seq method [47], as shown in Figure 1A,B and outlined in Table 2. In this way, the cell type-specific regulatory regions can be mapped in wide variety of cells. These have been deposited and curated in publicly available databases such as ENCODE [73], IHEC consortium [74], Ensembl [75], GTex [76] and Regulome DB [77], among others, which contain reams of information on the regulatory and structural elements of the genome for a number of cell and tissue types. Here, GWAS-implicated SNPs can be co-localised with cell type-specific regulatory regions to obtain an idea of the most likely causal SNP and cell type in a disease. Other publicly available resources, such as RNA expression data related to particular SNPs, regarded as expression quantitative trait loci (eQTL) [78], and the study of physical DNA/DNA interaction analysis via chromosome conformation capture (3c) techniques [79], can also indicate the gene that may be implicated with the risk variant. These data are extremely valuable but are available in a limited number of cell types, stimulatory conditions, and chronicity. Therefore, experimental validation is also necessary.

As mentioned earlier, ChIP-seq is commonly used to identify specific regulatory elements. For example, antibodies against histone modifications such as H3K4me1, H3K4me2 and H3K4me3 identify enhancers, the start of actively transcribed genes, and promoters, respectively [80]. A study from Farh et al. mapped SNPs from 39 GWASs across 21 autoimmune diseases to in-house generated RNA-seq and CHIP-seq data for H3K27ac, a marker of active promoters and enhancers [68]. This identified that the autoimmune diseases analysed showed a preferential risk of SNP enrichment in the enhancers of CD4+ cells. More specific to psoriasis, a study by Lin et al. [81] attempted to identify disease-relevant cell subtypes by analysing genetic variants from multiple GWASs in 26 types of human skin and immune primary cells and cell lines from the NIH Roadmap Epigenomics Consortium [82]. They then overlapped this with publicly available histone marker data, which was used to identify active enhancers. From this, they determined that 654 of 1609 SNPs in LD with key psoriasis SNPs were in active enhancers in CD4+ T-cells. Similarly, Tsoi and colleagues’ meta-analysis of psoriasis GWASs in European populations identified psoriasis SNPs enriched in enhancers unique to specific CD4+ subsets and CD8+ T cells [12]. These techniques enable the study of SNPs in their active cell type, providing the relevant biological conditions for fully capturing the action of these genetic perturbations.

## 7. Chromosome Conformation Capture and eQTLs

Advances in functional genomics techniques have enabled the study of the three-dimensional structure of the genome in incredible detail. Linear DNA is compressed and folded to create loops, enabling the physical interaction of genetic elements thousands of kilobases away. Chromosome conformation capture techniques such as 3C, 5C and most recently, Hi-C detect chromatin structures in their native state, as shown in Figure 1C. As described in Table 2, CHi-C goes one step further, using an intermediate hybridisation step to capture specific regions, such as promoters or enhancers, enriching and increasing the resolution of these areas of interest [48]. Developing these techniques was essential to understanding the changes in the structure and interactions of the genome caused by GWAS SNPs [83].

Ray-Jones et al. used psoriasis-focused CHi-C to link GWAS-identified psoriasis-associated variants with their target genes [84]. They identify chromatin interactions between the *KLF4* promoter and psoriasis-associated SNPs in distant enhancers. These interactions were identified in a skin cell line (HaCaT) but not in immune cells (My-La), indicating a cell type-specific control of this gene in psoriasis risk. Despite being over 500 kb away from the lead GWAS SNP, the interaction of the SNP with the promoter of *KLF4* leads to the upregulation of this transcription factor and its downstream effects on immune cell regulation and skin barrier function. 

In a similar study, Shi et al. combined public datasets from lymphoblastoid cell lines and primary CD4+ T cells with in-house generated HiChIP in HaCaT and My-La cell lines [85], linking GWAS variants to genes. Here, 52% of eQTL interactions were strengthened as true positive signals with corresponding physical interactions. This accurate HiChIP analysis identified MyLa-specific, long-range interactions between the intronic SNP rs9504361 and the promoter of *IRF4*. *IRF4* is a known psoriasis-associated transcription factor upregulated in psoriatic lesions.

## 8. Examining Phenotypic Differences Using Advanced Functional Techniques

Recently, CRISPR genome editing has revolutionised functional genomics and the possibilities in genomic medicine (Table 2). This system harnesses an RNA-driven bacterial defence mechanism aimed at recognising and cutting DNA sequences [16]. Breaks created by this machinery allow for the alteration, deletion, or addition of genetic sequences through the cell’s natural repair mechanism [16]. CRISPR offers precision in editing genetic sequences, potentially transforming clinical applications across diseases [17,18], including skin diseases. The CRISPR technology is also driving the understanding of how genetic risk variants act in disease in experimental models. By employing a modified, catalytically inactive version of the CRISPR enzyme, known as “dead Cas9” [86], it is possible to attach activators or repressors of transcription to target sites and assess the outcome on cellular phenotype. Furthermore, the gene regulatory machinery can target precise points in the genome. In this way, it is possible to turn on/off the GWAS-implicated regulatory switches and empirically confirm their gene target, the cell type in which they act and their downstream effect on gene transcription and cell function [86].

In one such experiment, Ray-Jones and colleagues harnessed the CRISPR technology to functionally investigate psoriasis-associated risk loci using HaCaT keratinocytes cell lines edited with CRISPR activation and inhibition of the regulatory region to validate a long-distance interaction between a psoriasis-associated locus and the *KLF4* gene [84].

As mentioned earlier, validation of psoriasis gene targets has also been studied in CRISPR knock out studies. The knock out of the gene *Zdhhc2* protected mice from developing disease upon treatment with imiquimod, protecting against local inflammation in the mice [87]. Moreover, a cross-disciplinary technological strategy harnessing the CRISPR approach for the therapeutic treatment of inflammatory skin disorders used a dissolvable microneedle patch for the transdermal co-delivery of glucocorticoids and genome-editing agents [88]. This system features nanoformulations of polymer-encapsulated CRISPR-Cas9 machinery targeting the NLRP3 inflammasome, a critical cell pathway, alongside dexamethasone nanoparticles [88]. Upon application, the MN patch dissolves in the skin, allowing the nanoformulations to be internalised by keratinocytes and surrounding immune cells [88]. This process facilitates targeted intervention in inflammation, illustrating a promising approach for treating inflammatory skin conditions [88]. While potential in dermatological sciences remains largely untapped, CRISPR-based trials are promising approaches in different fields—e.g., haematology, oncology, and infection.

## 9. Organoids

To facilitate precision medicine and better modelling of disease pathogenesis, the CRISPR method has been used in organoids, regarded as self-organised 3D tissue cultures [89]. Organoids represent a novel model system in the study of human diseases, due to their similarity with their tissue counterpart in terms of cell complexity and heterogeneity [90]. Boonekamp and colleagues generated murine-derived skin organoids by preserving their histological architecture—i.e., the typical basal-apical structure—while ensuring long-term and genetically stable expansion within a controlled in vitro system [19]. Interestingly, leveraging the CRISPR-mediated genome editing, the desmoplakin gene—a desmosome protein described for its role in skin pathologies [20]—was excised from epidermal organoids with significant perturbations of desmosomal organisation [19]. These advances highlight how skin organoids and CRISPR can be used as sophisticated models to study gene function and disease pathology in vitro—resulting in an ideal system for genetic manipulation offering a direct phenotypic evaluation.

## 10. Epigenetic Studies in Psoriasis

Applying multiomic approaches in skin biology—i.e., genomics, transcriptomics, proteomics, metabolomics, and microbiomics—is an unparalleled tool to gain profound insights into cutaneous physiopathology, thereby facilitating the development of precise, bespoke diagnostic and personalised therapeutic strategies in the future [91]. 

Epigenetic studies examine the reversible and heritable mechanisms of modifications in gene function—not involving alterations in the DNA sequence itself [92], but rather chromatin modifications that can modify the expression of genes. Evidence suggests that epigenetics can alter disease susceptibility in psoriasis. DNA methylation, associated with epigenetic gene silencing, involves methyl groups covalently bound to cytosines at CpG islands in the gene regulatory region [93] through a process catalysed by DNA methyltransferases (DNMT). DNA methylation is crucial in the regulation of time- and tissue-specific gene regulation. Different studies have pointed out the alteration in DNA methylation in psoriatic lesions when compared to healthy skin [94,95,96]. Differences in CpG methylation between psoriatic lesions and healthy skin were identified by Roberson and colleagues in a genome-wide study, identifying 1108 distinct sites, 12 of which were associated with genes significantly overexpressed in psoriatic skin samples [94]. Zhang et al. reported an increase in overall methylation in psoriatic skin and blood cells of patients affected by psoriasis—with upregulation of *DNMT1* and suppression of two relevant methyl-DNA binding domain genes *MBD2* and *MeCP2* in blood [95]. As described earlier, Li and colleagues demonstrated an epigenetic regulation of *IL23* levels, in keratinocytes, upon histone methylation (H3K9me2), which is crucial in driving psoriatic conditions, in mice [97]. Mounting evidence correlates histone methylation patterns and patient response to biologic therapies in psoriasis [98].

In conclusion, studying the methylation pattern of DNA is pivotal in studying complex biological mechanisms such as the ones that go awry in psoriasis. Therefore, two main methodologies could be mentioned. One common approach to determine DNA methylation is bisulphite genomic sequencing, which identifies methylation patterns at the single base pair resolution based on the different outcomes of chemical reactions of cytosines or modified cytosines in the presence of sodium bisulphite [21]. Another approach used to study epigenetic patterns is using methylation-dependent restriction enzymes that catalyse the excision of DNA fragments containing the modified cytosine. The latter enzymatically processed products can then be sequenced to determine epigenetic modifications in a specific study [99]. As the epigenetic reprogramming is pivotal in the development of psoriasis, using these tools is currently helping the scientific community to better elucidate the crucial role of epigenetics in triggering the disease [100].

## 11. Mouse Models

As reviewed by others [101], naturally occurring spontaneous mutations in murine models mirror certain features of psoriasis. These models include the chronic proliferative dermatitis model or the flaky skin mutant—which exhibits traits resembling psoriasis—and the Asebia mutants characterised by, among others, signs of the thickened dermis, increased angiogenesis, and alopecia [102]. As opposed to these mutation models, the xenograft models—consisting of transplanting human skin onto an immunodeficient mouse—allowed the identification of T cells as pivotal regulators of the immunopathogenesis aspects of psoriasis. Due to several technical difficulties, xenograft models are not commonly used to study psoriasis. As indicated by Gangwar et al., leveraging the CRISPR technology through genome editing led to the creation of transgenic murine models to study psoriasis [103]. CRISPR offers the great technological advantage of being able to flexibly modulate genes over time or in specific tissues—in either a cell- or lineage-specific manner—through the overexpression or suppression of specific gene targets in murine models. Indeed, upon a CRISPR-mediated specific removal of the *IL23A* gene in murine keratinocytes, Li et al. highlighted an epigenetic N-WASP-mediated repression of *IL23* initiated by TNF [97]. Using murine models to study regulative elements controlling genes involved in psoriasis-associated pathways would be instrumental in interrogating functional altered pathways in psoriasis, as previously performed in other diseases. For example, a 70-kilobase deletion on chromosome 4 impacted the expression of nearby genes in mice. These findings indicate a potential implication in coronary artery disease progression [104]. As also used in studying other immune conditions [105], humanised mouse models—where human DNA sequences, cells, tissues, or tumours are carried by a modified murine model—would significantly contribute to a better understanding of the disease [106].

## 12. Towards Novel Psoriasis Therapeutics 

Before the age of genomic medicine, non-specific treatments such as phototherapy, steroids, and the chemotherapy drug methotrexate were the only available therapies to treat psoriasis [107]. By conducting GWASs on large heterogeneous psoriasis cohorts and combining this with the information gleaned from functional genomics studies, therapeutics can be rationally designed, and current drugs repurposed to treat psoriasis, such as the use of IL-23 inhibitors [108]. These studies will also help clinicians predict patient response to therapy based on their genotype [23].

Drug repurposing is an effective approach to therapeutic development for both consumers and the pharmaceutical industry. Repurposing speeds up development time, and reduces the need for extensive pre-clinical testing, reducing costs. In a study by Jeong and colleagues [22], potential causal genes and tissue types were identified, revealing multiple genes with pre-existing drugs targets that had not previously been picked up for use in psoriasis. A total of 75 of the identified psoriasis-associated risk genes were inputted into the Drug Gene Interaction database [24]. One of the genes identified in this study was *ERAP1*, which generates the causal antigen presented by the PSORS1 gene HLA-C*06:02 involved in the autoimmune response against melanocytes [25]. It can be targeted with Esculetin, which is already used as a herbal treatment in Asian medicine. Esculetin is already known for its anti-inflammatory and antioxidant properties, and has been used in studies on colon carcinoma cell lines [26]. In murine psoriatic skin models, Esculetin specifically targets the NF-KB pathway and reduces the detectable mRNA levels of pro-inflammatory cytokines [109]. A similar study by Nanda et al. examined the NHGRI-EBI GWAS catalogue and integrated genomic, transcriptomic, and proteomic functional annotations using the GeneCards database [110]. Using the CanSAR database, druggable targets of significant psoriasis-associated genes were evaluated for any potential associated toxicities [27]. The two most promising targets were POLI and IL-13, with 58 and 2 FDA-approved drugs, respectively. SNPs in *IL13* are associated with an increased risk of developing PsA in psoriasis patients [111]. This discovery highlights how genetics screens, combined with functional annotation data, may reveal how drugs can be repurposed to treat psoriasis.

The growth of artificial intelligence (AI), including Machine Learning (ML), can also assist in developing therapies for psoriasis patients. This approach removes the labour and time-consuming elements of drug repurposing. Several recent studies have used AI programs such as ChatGPT [112], deep learning method Knowledge Graph [113] and virtual screening network ChemAI [114] to identify and validate potentially repurposable drugs. A recent ML study by ENSEMBL used over 30 databases to predict over 37,000 unknown drug–drug interactions in psoriasis [115]. Despite this, ML and AI cannot replace clinical efficacy and safety tests that must be performed before repurposed drugs can be used in the clinic. Indeed, understanding how AI can inform functional genomics-driven data—i.e., gene expression patterns from complex datasets—could help clarify and determine genetic markers to simulate and predict drug targets’ efficacy and potential toxicity [116]. 

Functional genomics has also identified genes that could be targeted therapeutically with the CRISPR-Cas9 system. In November 2023, the UK approved the world’s first CRISPR-Cas9 gene editing therapy for sickle cell disease and transfusion-dependent β-thalassemia. However, unlike previously discussed treatments, this intends to cure disease rather than manage symptoms [117] and it could potentially be applied to psoriasis. Wan and colleagues report a potential microneedle patch to deliver CRISPR-Cas9 machinery and RNPs targeting *NLRP3* [88]. Multiple GWASs have pointed towards this inflammasome gene as having several risk SNPs for psoriasis [118,119,120].

## 13. Conclusions

Psoriasis is a chronic skin condition that causes periodic flare-ups and can significantly impact a person’s quality of life [121]. Existing therapies seek to control clinical symptoms, rather than provide a cure, which imposes significant burdens on society and family structures. The exact cause of this skin condition is not yet fully understood. However, it is likely that a combination of genetic, environmental, and immunological variables play a significant role in disease aetiopathogenesis, as suggested by psoriasis risk loci overlapping genes involved in maintaining the skin barrier function, innate and adaptive immune responses [121]. 

As mentioned earlier, variants in the *LCE* cluster and GJB2 have a significant impact on disease susceptibility given their role in the stability of keratinocytes and cutaneous differentiation [122,123]. Importantly, mutations in NF-κB signalling pathway-associated proteins have been shown to cause psoriasis [124]. Furthermore, it is worth noting that alterations in genes involved in the IFN pathway have also been implicated in cutaneous diseases [57]. As described, altered activation of the adaptive branch of immunity, specifically T cells—together with the involvement of the IL-23/17 axis—characterises an aberrant Th17-driven disease. In this context, genetic alterations have a role in altering normal antigen presentation or T-cell functionality [125]. Interestingly, dysregulation of the *SOCS1* gene, which codes for a key molecule involved in the IFN pathway and Th17 differentiation, highlights the complexity of aberrant immune responses that have a role in psoriasis susceptibility and the genetic similarity with other chronic conditions such as Crohn’s disease [56,125,126].

This review aims to provide the most recent and commonly used approaches in functional genomics for complex diseases and particularly in psoriasis. Despite our best efforts to provide a comprehensive overview of this topic, it is important to note that this review is not systematic. As a result, it may not cover all relevant publications. Nevertheless, this review presents the advancements in omics technologies that are improving the accuracy of genetic target identification while propelling the development of more refined and targeted treatment strategies. Functional genomics has been instrumental so far in linking genes with conditions and the biological impact of genetic alterations—mainly those found in non-coding genomic regions—that are likely to regulate gene expression and have important phenotypical consequences. By uncovering regulatory elements, through the in-depth analysis of these latter regions, it is possible to develop the field of precision medicine, paving the way to targeted treatment therapies that could also cure the disease and have a huge societal impact. 

## Data Availability

Not applicable.
